# Characterization of the effect of histone deacetylation inhibitors on CD8^+^ T cells in the context of aging

**DOI:** 10.1186/s12967-022-03733-9

**Published:** 2022-11-22

**Authors:** Georgiana Toma, Eliza Karapetian, Chiara Massa, Dagmar Quandt, Barbara Seliger

**Affiliations:** 1 Institute of Medical Immunology, Martin Luther University Halle-Wittenberg, Magdeburger Str. 2, 06112 Halle, Germany; 2 Fraunhofer Institute for Cell Therapy and Immunology, 04103 Leipzig, Germany

**Keywords:** Histone deacetylase inhibitors, CD8^+^ T cells, Protein identification, Acetylation, Aging

## Abstract

**Background:**

Posttranslational protein modifications regulate essential cellular processes, including the immune cell activation. Despite known age-related alterations of the phenotype, composition and cytokine profiles of immune cells, the role of acetylation in the aging process of the immune system was not broadly investigated. Therefore, in the current study the effect of acetylation on the protein expression profiles and function of CD8^+^ T cells from donors of distinct age was analyzed using histone deacetylase inhibitors (HDACi).

**Methods:**

CD8^+^ T cells isolated from peripheral blood mononuclear cells of 30 young (< 30 years) and 30 old (> 60 years) healthy donors were activated with anti-CD3/anti-CD28 antibodies in the presence and absence of a cocktail of HDACi. The protein expression profiles of untreated and HDACi-treated CD8^+^ T cells were analyzed using two-dimensional gel electrophoresis. Proteins with a differential expression level (less than 0.66-fold decrease or more than 1.5-fold increase) between CD8^+^ T cells of young and old donors were identified by matrix-associated laser desorption ionization—time of flight mass spectrometry. Functional enrichment analysis of proteins identified was performed using the online tool STRING. The function of CD8^+^ T cells was assessed by analyses of cytokine secretion, surface expression of activation markers, proliferative capacity and apoptosis rate.

**Results:**

The HDACi treatment of CD8^+^ T cells increased in an age-independent manner the intracellular acetylation of proteins, in particular cytoskeleton components and chaperones. Despite a strong similarity between the protein expression profiles of both age groups, the functional activity of CD8^+^ T cells significantly differed with an age-dependent increase in cytokine secretion and expression of activation markers for CD8^+^ T cells from old donors, which was maintained after HDACi treatment. The proliferation and apoptosis rate of CD8^+^ T cells after HDACi treatment was equal between both age groups.

**Conclusions:**

Despite a comparable effect of HDACi treatment on the protein signature of CD8^+^ T cells from donors of different ages, an initial higher functionality of CD8^+^ T cells from old donors when compared to CD8^+^ T cells from young donors was detected, which might have clinical relevance.

**Supplementary Information:**

The online version contains supplementary material available at 10.1186/s12967-022-03733-9.

## Background

It is well established that a shift from a predominantly naïve to a more diverse memory T cell phenotype occurs during aging [1–3]. This coincides with an increase in overall inflammation [4] and a reduced vaccination efficacy in older individuals [5, 6]. Furthermore, post-translational modifications (PTMs), like acetylation and phosphorylation, not only affect T cell functions, but might also influence the incidence of diseases during aging [7–10]. While phosphorylation is mostly involved in cellular signaling, acetylation has a wide range of substrates resulting in the regulation of gene expression, DNA binding, mRNA and protein stability, interactions and localization [11–14]. Acetylation is regulated by acetyltransferases (i.e., p300), which transfer an acetyl group to lysine residues in proteins, and histone deacetylases (HDAC), which remove it. HDACs include the NAD^+^-dependent sirtuins (SIRT 1–7) as well as the Zn-dependent deacetylases (HDAC 1–11) [14]. Both enzyme classes are key epigenetic regulators of gene and protein expression and are involved in the dynamic changes of histone acetylation.

One role of Zn-dependent HDACs is the control of longevity and the regulation of physiologic processes in immune cells. For example, HDAC3 is crucial for the T cell development after hematopoietic stem cell commitment to the T cell lineage [15], T cell post-thymic maturation [16] and acts as an inhibitor for the cytotoxicity of CD8^+^ T cells after infection clearance [17]. HDAC11 regulates the inflammatory response of T cells, which is confirmed by HDAC11 deficient mice exerting a higher inflammatory activity [18]. Furthermore, an increased expression of several HDACs was found in numerous diseases, which was associated with aging including different cancer types, such as e.g., acute lymphoblastic lymphoma [19–21], prostate, breast and colorectal cancer [22].

Unlike genetic changes, histone acetylation is reversible and thus represents an attractive therapeutic target. Indeed, HDAC inhibitors (HDACi) are one of the most promising agents in anti-aging research. In addition, different HDACi have been shown to have clinical potential and are currently used for the treatment of many chronic pathological conditions due to their anti-inflammatory and anti-cancerous properties [23–26]. These include trichostatin A (TSA), a pan-inhibitor of Zn-dependent HDACs [27], which also interacts with SIRT6 [28], and EX-527, a specific inhibitor of SIRT1 [29]. EX-527 has anti-inflammatory properties and impairs T cell activation [30]. Another inhibitor with anti-inflammatory activity is nicotinamide (NAM), which is the physiologic inhibitor of sirtuins [31, 32]. These HDACi have been used in vitro and in vivo in experimental models and/or in humans to modulate HDAC activity and to determine the role of acetylation in physiologic and pathophysiologic cellular processes. The modification of the acetylation and deacetylation status of signaling molecules and transcription factors by classical HDACs provides the rational for the treatment with HDACi, which have been already approved for clinical use [33, 34]. However, there exists little information about the acetylation status of proteins in cancer patients during treatment.

Stimulation of CD8^+^ T cells in vitro using antibodies directed against the T cell receptor (TCR) and CD28 resulted in an activation of multiple proteins involved in the TCR signaling cascade including the serine-threonine-kinases [35], such as e.g. protein kinase B (AKT), which plays a major role in proliferation, apoptosis and glucose metabolism of activated T cells [36]. After TCR activation and CD28 co-stimulation, AKT is phosphorylated either by the mTOR complex 2 (mTORC2) or by other proteins [36] leading to its activation and entry into the nucleus, where it phosphorylates the fork head box O (FoxO) transcription factors resulting in their inactivation. The FoxO proteins are more active in quiescent naïve T cells promoting the expression of molecules like L-selectin (SELL or CD62L) and C–C chemokine 7 (CCR7) [35]. AKT is also essential for changes in the metabolism of memory T cells with a transition to glycolysis during activation [37] and for the early metabolic reprogramming following naïve T cell activation [38].

Acetylation changes have broad effects on the cellular metabolism [39, 40] by transcriptional control of certain genes and modification of key proteins, such as glyceraldehyde-3-phosphate dehydrogenase (GAPDH). Acetylation of GAPDH results in its activation and regulation of its localization [41, 42]. Furthermore, many proteins involved in the tricarboxylic acid cycle [11] as well as cytoskeletal components, such as tubulin [43], actin and cortactin [44, 45], are acetylated. There exists also increasing evidence that HDACi decrease immune suppression and enhance immune cell function including that of T cells [46].

However, the role of acetylation in age-dependent immune responses has not yet been investigated in detail. Since the interplay between both processes is insufficiently understood, the aim of this study was to determine the effect of acetylation on the proteome and function of CD8^+^ T cells in the context of aging.

## Methods

### Biological material and T cell culture

Buffy coats from healthy young (< 30 years) and old donors (> 60 years) were obtained from the blood bank [47] of the University Hospital of the Martin Luther University Halle-Wittenberg in Halle (Saale), Germany, upon informed consent of the donors. In total, blood samples from 30 young and 30 old donors were used, and isolated cells were distributed among the current assays. Peripheral blood mononuclear cells (PBMC) were separated using the density gradient method with Bicoll (1.077 g/ml, Biochrom, Berlin, Germany). CD8^+^ T cells were magnetically sorted with CD8^+^ beads (Miltenyi, Bergisch Gladbach, Germany) according to the manufacturer’s protocol. Only preparations with a purity of CD3^+^CD8^+^ T cells over 95% were employed for further analyses. The CD8^+^ T cells were seeded at a density of 1 × 10^6^ cells/ml and cultured for the indicated time in X-Vivo15 medium (Lonza, Verviers, Belgium) supplemented with 1% (v/v) L-glutamine (Lonza) and 1% (v/v) PenStrep (Sigma, Darmstadt, Germany). CD8^+^ T cells were activated with plated 2.5 µg/ml anti-CD3 monoclonal antibody (mAb; Thermo Fisher Scientific, Waltham, MA, USA) and soluble 1 µg/ml anti-CD28 mAb (BD Biosciences, San Jose, CA, USA). Non-activated and activated CD8^+^ T cells were only used for the analysis of the relative HDAC expression. For all other assays, activated CD8^+^ T cells were either left untreated or treated with the HDACi cocktail (Santa Cruz, Dallas, TX, USA, with a 1:250 dilution to a final concentration of 160 nM TSA, 4 µM Ex-527 and 1.6 mM NAM).

### Flow cytometric analysis

The purity of the sorted CD8^+^ T cells was assessed by staining of 10^5^ cells/donor post-sort with anti-CD3 FITC and anti-CD8 BV605 mAbs (BD Biosciences) using a standard protocol. To determine the activation of CD8^+^ T cells, 10^5^ CD8^+^ T cells were stained 48 h after stimulation [46] with anti-CD69 FITC, anti-CD25 PE (BD Biosciences) and anti-CD71 APC-H7 mAbs (Biolegend, San Diego, CA, USA). The percentages of cells expressing the activation surface markers were determined by gating the positive cells in the histogram of each fluorophore. Flow cytometric analyses was performed on a BD LSR Fortessa flow cytometer (BD Biosciences). Data were analyzed with the BD FACS Diva software (BD Biosciences).

### Expression analysis of transcripts

For mRNA expression analyses, CD8^+^ T cells upon the indicated treatment were washed once with ice-cold PBS, before adding the lysis buffer from the NucleoSpin RNA kit (Macherey–Nagel, Düren, Germany) supplemented with β-mercaptoethanol (AppliChem GmbH). Total RNA was isolated according to the company’s protocol and then subjected to quantitative PCR (qPCR). For each sample, cDNA was synthesized from 500 ng of total RNA using a RevertAid First Strand cDNA synthesis kit (Thermo Fisher Scientific). qPCR reactions were done using 2X SYBR Green qPCR Master Mix (Promega, Madison, WI, USA) according to the manufacturer’s recommendations using the primers listed in Additional file 1: Table S1. qPCR data are presented as ΔΔCq (fold change) calculated using the TATA-binding box protein (TBP) and succinyldehydrogenase subunit A (SDHA) as housekeepers according to the following formula: $\Delta \Delta Cq =\left(CqHK-CqGOI\right)treated-\left(CqHK-CqGOI\right)untreated$ [48]. CqHK represents the mean of the quantification cycle (Cq) values for the housekeepers (HK) and the CqGOI is the Cq of the gene of interest (GOI) in the indicated condition.

### Analysis of the relative protein expression

Total cellular proteins from CD8^+^ T cells were extracted and analyzed by Western blot (WB) as previously described [49]. Briefly, 20 µg of total protein/sample were separated on a 10% sodium dodecyl sulphate–polyacrylamide gel electrophoresis (SDS-PAGE) gel and then transferred to a nitrocellulose membrane (GE Healthcare, Uppsala, Sweden). The membranes were blocked for one hour with 5% bovine serum albumin and then incubated overnight at 4 °C with the primary mAbs directed against HDAC3, HDAC4, HDAC11, AKT, p-AKT, tubulin, acetyl-tubulin, 14-3-3 ζ/δ and the heat shock protein 90 (HSP90; all purchased from Cell Signaling Technologies, Danvers, MA, USA). After washing, the membranes were incubated for one hour with an anti-rabbit or an anti-mouse horseradish peroxidase-conjugated secondary antibody (Cell Signaling Technologies). The substrate used for detection of HDAC4 and tubulin staining was the Lumi-Light Western-Blotting Substrate (Roche), for all other samples the SignalFire ECL Reagent (Cell Signaling Technologies). The final chemiluminescence pictures were taken with a FujiFilm LAS-300 (FujiFilm, Tokyo, Japan). The fold change in protein expression was determined using ImageJ Lite software and normalized to the Ponceau staining of each membrane. Data are presented as a ratio between the signal of treated vs. untreated samples. Values of > 1 and < 1 represent an increase or a decrease in the expression of the protein after treatment, respectively.

### Determination of the cytokine secretion

After 48 h of culture with the indicated treatment, the supernatant of CD8^+^ T cells was removed and stored in aliquots at − 20 °C. The concentration of soluble proteins was determined using BD Cytometric Bead Array Flex Sets (BD Bioscience) containing IFN-γ, TNF-α, IL-3, IL-4, IL-5, IL-6, IL-10, granulocyte–macrophage colony-stimulating factor (GM-CSF), granzyme A and granzyme B according to the manufacturer's protocol. The results are presented as pg/ml or ng/ml for each sample type.

### Determination of the cell proliferation and apoptosis

For determination of the cell proliferation, CD8^+^ T cells were stained with 10 µM CellTrace™ Carboxyfluorescein succinimidyl ester (CFSE) Cell Proliferation Kit (Thermo Fisher) as described [50], then seeded at a density of 10^6^ cells/ml and cultured for five days. After this incubation, CD8^+^ T cells were washed once with PBS and directly analyzed by flow cytometry. The flow cytometric data were processed using FCS Express 6 Plus Research Edition (San Diego, CA, USA) with the software’s standard specification for proliferation assays. Results are presented as proliferation indexes indicating the average number of times the cells have divided.

For the determination of the apoptosis rate, CD8^+^ T cells either left untreated or treated for the indicated time were stained with an annexin-V kit (Miltenyi) according to the manufacturers’ protocol followed by flow cytometric analysis. Staining with a FITC labelled anti-annexin-V mAb and propidium iodide (PI) was used to discriminate between alive cells (annexin^−^, PI^−^), debris (annexin^−^ PI^+^), dead cells (annexin^+^ PI^+^) and apoptotic cells (annexin^+^ PI^−^).

### Two-dimensional gel electrophoresis (2DE) and isoelectric focusing

CD8^+^ T cells were cultured as previously described for 48 h, before protein samples were prepared for 2DE separation [51]. Ten µg cell lysate/donor were stained with difference gel electrophoresis (DIGE) dye (SERVA Lighting SciDye Set, Serva, Heidelberg, Germany), according to the manufacturer`s indications. The isoelectric focusing was done on an IPG strip of 3–10 pH (GE Healthcare). A total of 5 replicates/sample were performed for each group. Isoelectric focusing was done overnight to a total of 48,000 V using an ETTAN IPGphor 80-6414-02 IEF Isoelectric Focusing Unit (Amersham Pharmacia Biotech, San Francisco, CA, USA) with a current of 50 µA/strip. The proteins were then separated overnight at 100 V on 13% T/2.5% C gels. The analytical gels were scanned with a Fujifilm FLA-5100 fluorescent image analyzer (FujiFilm, Tokyo, Japan) and the 2Delta software (DECODON, Greifswald, Germany) was used for matching of gels. This allows to identify spots, which changed their expression upon treatment. The mean of the normalized spot intensity was calculated for each individual spot among the five biological replicates. The regulation factors (RFs) were calculated for each individual spot as a ratio between the mean of the normalized spot intensity of the untreated and treated CD8^+^ T cells and the untreated CD8^+^ T cells for each age group separately. Only spots with a RF < 0.66 or > 1.5 were subjected to protein identification with matrix-assisted laser-desorption/ionization time-of-flight mass spectrometer (MALDI-TOF-MS). The threshold for the RF represents an increase or decrease of the protein expression of at least 1.5-fold.

### Identification of differentially expressed protein

For protein identification, the protein lysate from 10 donors/group were pooled and 250 µg total protein were separated by preparative 2DE gels, which were stained with a Commassie dye solution (23.1% H_3_PO_4_, 10% (NH_4_)_2_SO_4_, 20% MeOH, 0.1% Commassie Brilliant Blue—all from Applichem) for 72 h and then scanned using Bio-5000plus (Microtek, Hsinchu, Taiwan). The relevant spots were determined by comparison with the 2DE DIGE analytical gels, then picked and digested using DigestPro MSi (Intavis) with Trypsin (Promega) prior to protein identification by MALDI-TOF-MS (ultrafleXtreme™, Bruker Daltonics Inc., Bremen, Germany) using the FlexControl Ultraflex Tof/Tof software (Bruker) with the Rp 700–3500 Da method. All samples were calibrated to the Peptide Calibration Standard II (Bruker). The background peaks were identified and eliminated employing the FlexAnalysis software and the resulting peak list was used to identify the proteins using Biotools 3.2 (both from Bruker), which employs the Matrix/Mascot database (Matrix Science, Dauhaim, USA).

### Network analysis and GO term enrichment

Identified, differentially expressed candidates were analyzed for network interaction using the online search tool for the retrieval of interacting genes (STRING) [52, 53] with a threshold for confidence of 0.7. The STRING functional protein association networks also perform gene ontology (GO) term enrichment analysis. The resulting GO terms were filtered for repetitions (e.g., “Cytoplasm” and “Cytoplasmic part”), redundancies (e.g., “leukocyte activation”, “leukocyte mediated immunity,” and “leukocyte activation involved in immune response”) and irrelevant terms (e.g., “Myelin sheath”). For each GO term, the strength and false discovery rate (FDR) was calculated. The overlap between the identified proteins found in different cellular compartments was calculated with Venny 2.0 [54].

### Statistical methods

The Mann–Whitney statistical test was employed and calculated using GraphPad Prism 9. For the 2D protein expression profiles, a t-test was performed with the 2Delta Decodon software. p values ˂ 0.05 are marked with *, p values ˂ 0.01 with **, p values < 0.001 with *** and p values ˂ 0.0001 with ****.

## Results

### HDAC expression and changes in the acetylation pattern of proteins

**Fig. 1:**
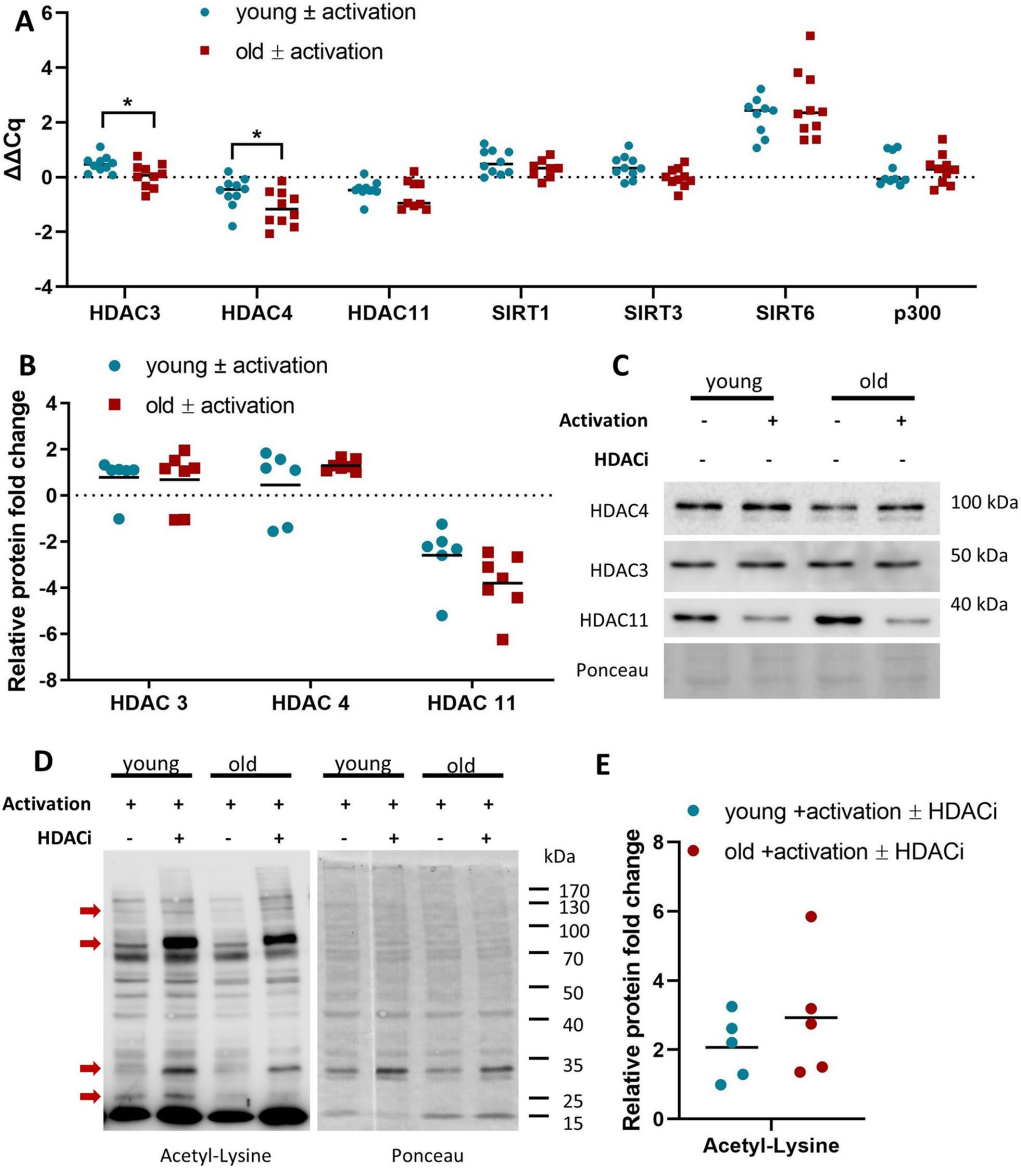
Effect of T-cell activation on the HDAC expression and acetylation profiles after HDACi treatment. **A** Relative gene expression of selected genes after 48 h of TCR-mediated activation of CD8^+^ T cells. Total RNA was isolated, and qPCRs were prepared for the selected HDACs, sirtuins and p300. SDHA and TBP are used as housekeepers. N = 10, *p value < 0.05. **B** Relative protein expression of selected HDACs after 48 h of TCR-mediated activation of CD8^+^ T cells. The relative protein expression is calculated as a ratio between the untreated and the treated cells and it is relative to the Ponceau total protein staining. N young = 6, N old = 7. **C** Representative blots for the selected HDACs. Two donors are presented for each condition and for all selected HDACs. **E** Relative expression of the acetylation profiles of activated CD8^+^ T cells after HDACi treatment. The relative expression of acetylated lysine was normalized to the Ponceau total protein staining and calculated as a ratio between untreated and HDACi treated samples. N = 5. **D** Representative acetylation blots. Two donors are presented for each condition. Red arrows indicate the bands with the most noticeable increase in acetylation

To ensure that any changes in the protein expression pattern were specifically induced by the HDACi treatment and not due to a pre-existing difference in the expression of HDACs, the relative constitutive protein and transcript expression of HDACs during T cell activation in the absence of HDACi treatment was determined. The effect of the CD8^+^ T cell activation on the gene expression of deacetylases was determined by qPCR. As shown in Fig. 1A, the relative gene expression of the selected deacetylases, HDAC3, 4 and 11, SIRT1, 3 and 6, as well as the acetyltransferase p300 was not significantly influenced by T cell activation with the exception of SIRT6. CD8^+^ T cells from both age groups exhibited comparable expression pattern of each enzyme. Although there were statistically significant differences between the two age groups for HDAC3 and HDAC4, this change was not biologically significant. Comparable results were obtained for the relative protein expression of these enzymes (Fig. 1B and C), whereas the relative HDAC11 protein expression was considerably decreased compared to the gene expression. Overall, no biologically significant differences were found between the cohorts for the expression of deacetylases in CD8^+^ T cells, but a two- to three-fold increase in the acetylation levels after HDACi treatment were detected with an outlier presenting a sixfold increase in CD8^+^ T cells from old donors (Fig. 1D and E).

### Differential protein expression profiles of activated CD8^+^ T cells from young and old donors

**Fig. 2:**
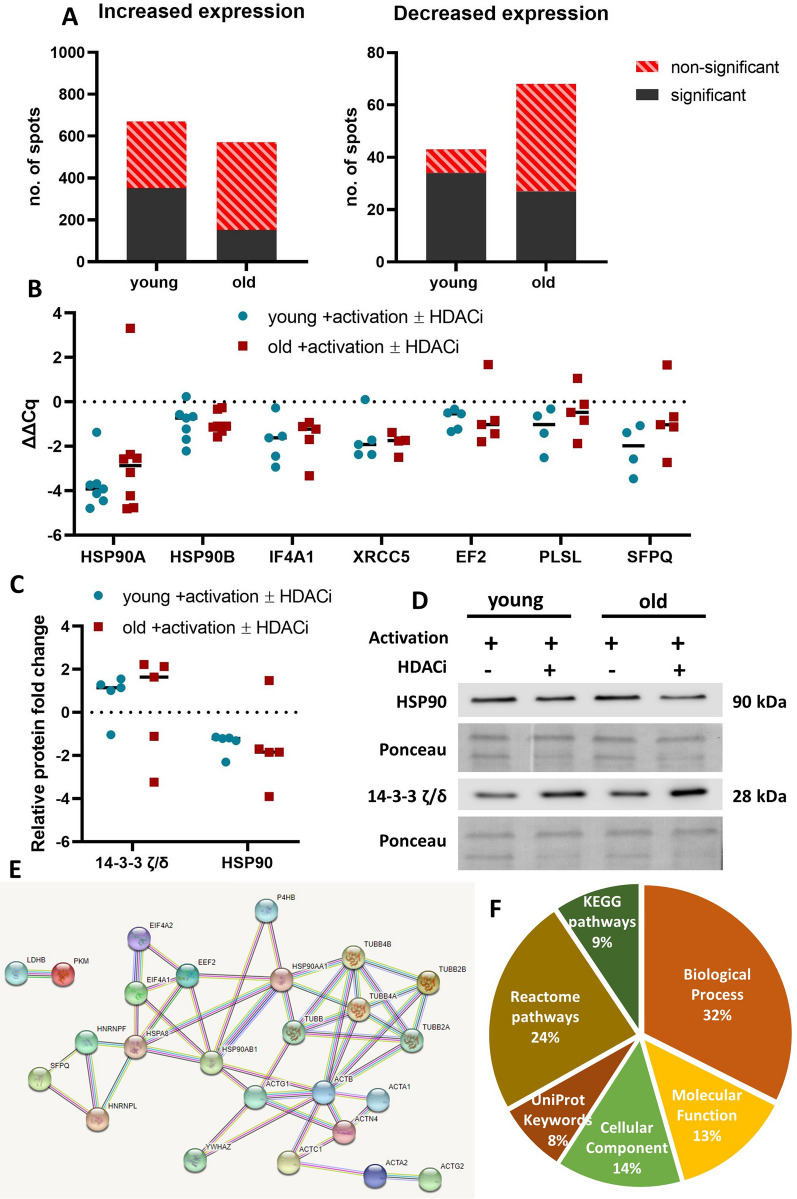
Protein expression, validation of identified proteins and protein interaction network. **A** Summary of protein spots as obtained by 2DE DIGE gels. Each protein spot has one RF calculated as a ratio between the mean fluorescence intensity of the HDACi-untreated samples versus that of the HDACi-treated samples for each age group separately. All the spots detected for young and old with an RF < 0.66 and > 1.5 were determined as biologically significant. These spots were further divided into spots with an increased expression (RF > 1.5) and with a decreased expression (RF < 0.66). Statistical significance was determined with a t-test and a p value < 0.05. **B** qPCR validation of identified proteins. The mRNA levels of selected proteins were measured with qPCR. The ∆∆Cq value is presented, and the housekeepers were TBP and SDHA. N = 4–8. **C** Relative protein expression of 14-3-3 ζ/δ and HSP90. The relative protein expression is presented as a ratio between the untreated and treated activated CD8^+^ T cells and it was normalized to the total protein staining with Ponceau S. N = 5. **D** Representative blots for the relative protein expression. Two donors are presented for each condition and for each protein. **E** STRING network analysis of the identified proteins. All the proteins identified were introduced into STRING protein–protein association network analysis online tool. The presented network was obtained with the option “multiple proteins” and a confidence of 0.7. **F** Overview of GO terms found for the identified proteins. The GO terms found were automatically divided into several categories. A total of 404 GO terms were returned and the proportion of each category is presented in a pie chart

Activated CD8^+^ T cells from young and old donors either left untreated or treated with an HDACi cocktail were subjected to proteome analysis using the 2DE DIGE method. Employing this approach, a total of 1503 differentially expressed protein spots were detected in CD8^+^ T cells from young and 1,484 in CD8^+^ T cells from old donors. Among them, 709 protein spots from the young and 658 from the old donors were considered biological significant (RF < 0.66 and > 1.5) with 387 and 180 reaching a statistical significance in the young and old cohort, respectively. From the biologically significant protein spots, the number of spots with an increased expression was larger for both age groups (Fig. 2A). Interestingly, CD8^+^ T cells of old donors had more spots with a decreased protein expression than that of young donors, but the statistical significance of the regulated spots was reduced for the older group suggesting an increased heterogenic protein abundance within this group.

Significantly modified spots of both age groups were selected for MALDI-TOF identification. Twenty-two out of the approximately 1.500 spots detected with the DIGE dyes were identified as listed in the Additional file 1: Table S2 together with their RFs. Multiple proteins were identified in several spots, which is a limitation of the method used [55]. The identified proteins presented a similar trend in the expression levels for both age groups with a general decreased expression after HDACi treatment (82.8% of the identified proteins had a decreased expression). A similar trend was found for the relative gene expression of these proteins (Fig. 2B).

The elongation factor 2 (EF2) and the tRNA pseudouridine synthase 1 (TRUB1) are the only two proteins identified that were differentially expressed in CD8^+^ T cells from old donors upon HDACi treatment but were unaffected in CD8^+^ T cells from young donors. Conversely, twelve proteins with an altered expression pattern were found in young donors, which were unchanged in old donors, including actin and tubulin.

The highest increase in expression of all proteins identified was found for the 14-3-3 ζ/δ protein with a RF of 4.82 for the young (p value < 0.0001) and RF of 3.17 for the old (p value < 0.01) donors. The heat shock protein 90 alpha (HSP90A) was identified in 2 different spots with a reduced expression after HDACi treatment with a RF of 0.47 for the young (p value < 0.05) and RF of 0.42 for the old donors (p value < 0.01). However, the WB data only partially confirmed the expression of the 14–3-3-ζ/δ and HSP90 proteins (Fig. 2C and D) with an approximately twofold decrease in both age groups.

### Functional protein association network analysis of identified proteins

All proteins identified were subjected to network analysis using the STRING online tool. An interaction network was found for some of the input proteins with a protein–protein interaction enrichment (p value of 10^–13^), indicating an increased interaction and participation in shared functions of these proteins (Fig. 2E). Three clusters were formed in the network: the first cluster included the heat shock proteins, the eukaryotic initiation factors, the elongation factor and splicing factor, while the two other clusters were centered around tubulin and actin, respectively. The 14-3-3 ζ/δ protein was only connected to actin. The proteins not interacting with any protein are the Rho GTPase activating protein 39, tubulin beta-3 chain, vimentin, tyrosine-protein phosphatase non-receptor type 6 (PTN6), X-ray repair cross-complementing protein 5, plastin-2,3-hydroxyacyl-CoA dehydrogenase type 2 and TRUB1.

**Fig. 3:**
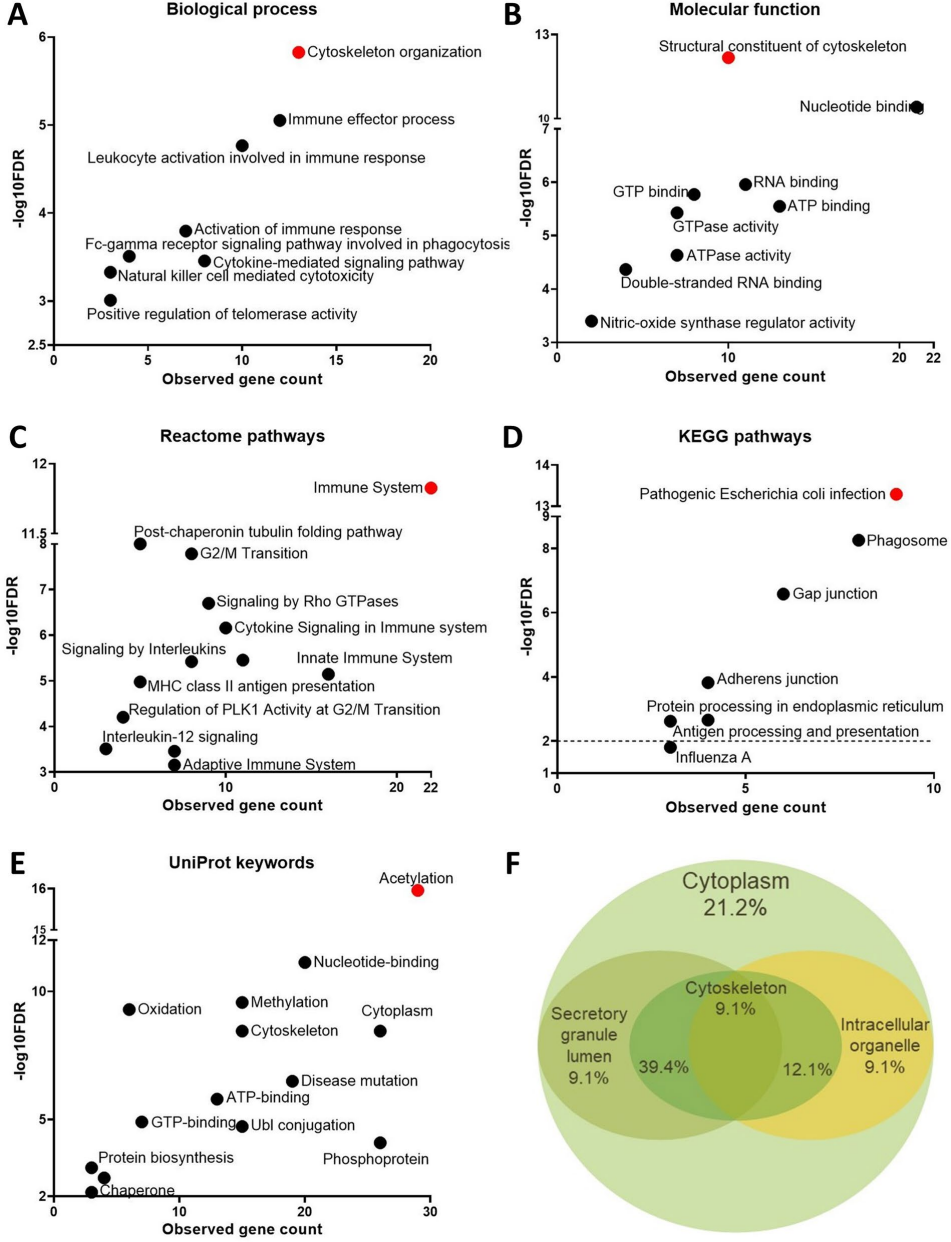
Go term enrichment for the differentially expressed proteins between CD8^+^ T cells from young and old donors. The STRING output is organized in several categories: Biological process (**A**), Molecular function (**B**), Reactome pathways (**C**), KEGG pathways (**D**) and UniProt keywords (**E**). Each GO term has XZ coordinates corresponding to the inverse of the False Discovery Rate (FDR), which describes the statistical significance, and the observed gene count, which represents the number of proteins from the input, which are annotated with the respective GO term. A dotted line is placed at x = 2 which corresponds to an FDR of 0.01, when necessary. The terms with the lowest FDR are colored red. **F** Cellular compartment distribution. The Venn diagram represents the GO terms found by STRING for the Cellular Component category. The proportions of each compartment are calculated with Venny 2.0

Next to network analysis, STRING automatically performs GO term enrichment using all input proteins. The resulting GO terms were divided into the categories biological process, molecular function and cellular component. Additionally, STRING searches other databases and compiles a list of pathways or keywords that are associated with the input proteins. Using this tool, 404 terms were identified (Fig. 2F and Additional file 1: Table S3) with the highest number for the biological process category (32%) followed by the Reactome pathways (24%). As shown in Fig. 3, multiple terms relating to immunity and T cells were identified. Several terms within the Kyoto encyclopedia of genes and genomes (KEGG) and Reactome pathways were related to the immune response (e.g., “influenza A”) or describing the immune system (e.g., “adaptive immune system”). For the Reactome pathways, the “immune system” term had the lowest FDR and the highest number of proteins in the network. Another focal point of the GO terms is the cytoskeleton, since the terms with the lowest FDRs in the biological process and molecular function categories were “cytoskeletal organization” and “structural constituent of the cytoskeleton”, respectively. Furthermore, the cytoskeleton is overlapping with the secretory granule lumen and the intracellular organelles with all input proteins located in the cytoplasm (Fig. 3F). In total, 39.4% of proteins were shared between the secretory granule lumen and the cytoskeleton, 21.2% proteins were solely in the cytoplasm.

Of the UniProt keywords, which mainly describe general roles or modifications of the input proteins, “acetylation” was the keyword with the lowest FDR and defined 29 proteins in the network. Out of 35 proteins identified, only four proteins were not defined by acetylation. These were the eukaryotic initiation factor 4A-II, tubulin beta-4A chain, tubulin beta-3 chain and PTN6. Thus, the data can be considered as an additional validation regarding the HDACi treatment used.

### Influence of HDACi treatment on structural components, signal transduction and differentiation of CD8^+^ T cells

**Fig. 4:**
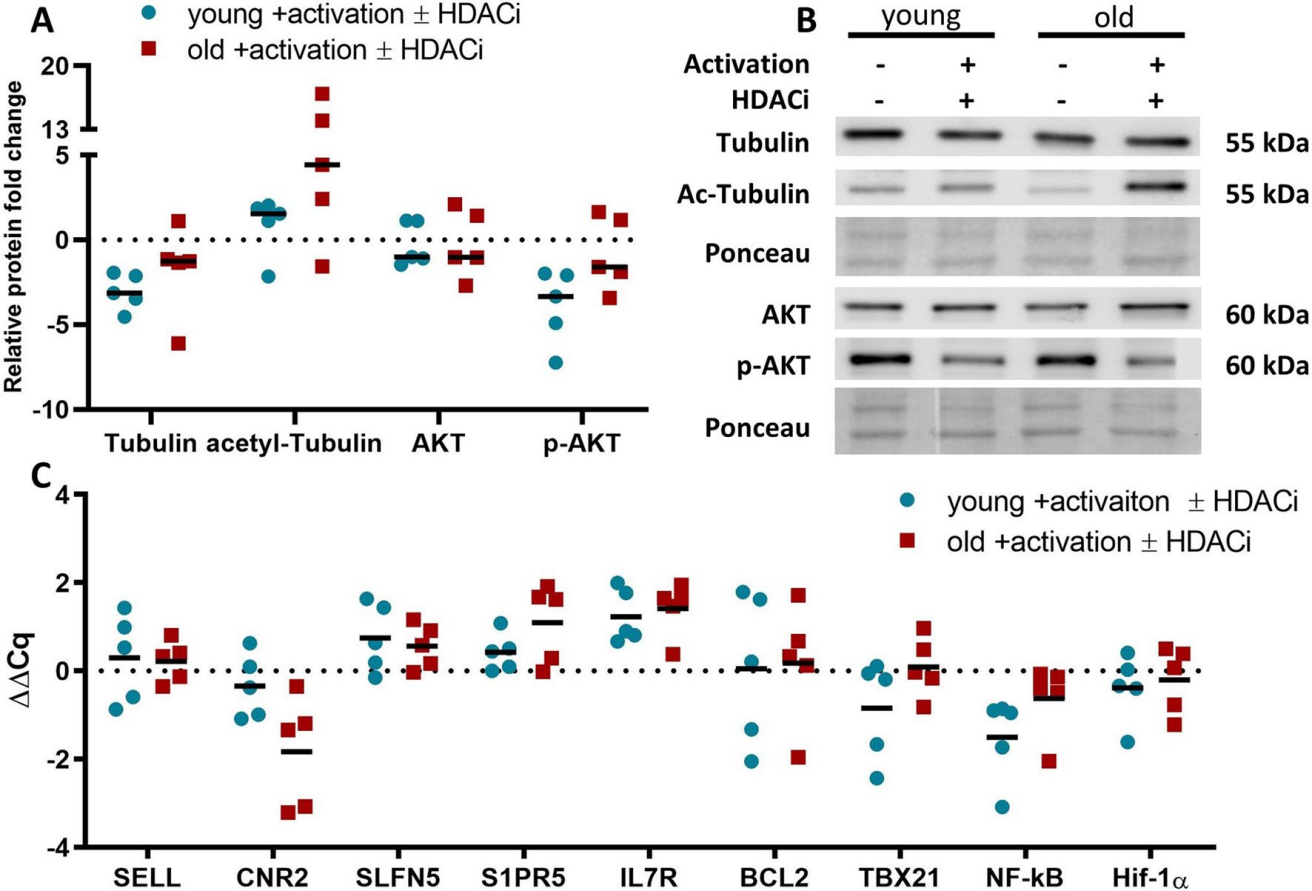
Relative protein and gene expression of selected genes. **A** Relative protein expression of selected proteins. The relative protein expression is presented as a ratio between the untreated and treated activated CD8^+^ T cells and it was normalized to the total protein staining with Ponceau S. For the modified proteins, an additional normalization was done to the unmodified protein. N = 5. **B** Representative blots for the relative protein expression. Two donors are presented for each condition and for each protein. **C** Relative transcript expression of genes involved in CD8^+^ T cell differentiation. The relative transcript expression of certain genes was calculated as described before and normalized to the same housekeepers. N = 5

To determine the effects of the HDACi treatment on the signal transduction, the relative protein expression pattern of tubulin, acetyl-tubulin, AKT and p-AKT was analyzed in CD8^+^ T cells of young and old donors by WB. The protein expression for both tubulin and AKT did not significantly differed between both age groups. However, while AKT expression was not modulated by HDACi treatment, tubulin expression was slightly decreased, with a more reduced expression in young donors (Fig. 4A and B). Acetyl-tubulin was increased after the HDACi treatment in CD8^+^ T cells independent of age with one outlier with a decreased expression in each group. Despite the difference in the expression pattern between young and old donors was not statistically significant, the increase in the expression of CD8^+^ T cells from old donors ranged from 4- to 19-fold, whereas it was around threefold for CD8^+^ T cells from young donors. The expression of p-AKT was reduced after HDACi treatment, which was more pronounced in CD8^+^ T cells from young donors.

In addition, selected genes involved in T cell differentiation [56, 57] were analyzed for their mRNA expression levels (Fig. 4C). All transcripts presented a heterogeneous expression pattern with no significant differences in CD8^+^ T cells between both age groups and not exceeding a twofold change. The cannabinoid receptor 2 (CNR2), which is involved in the secretion of pro-inflammatory factors [58], showed a minimal, not statistically significant difference between the age groups with a stronger decrease in CD8^+^ T cells of old donors.

### Reduced expression of activation markers and its association to proliferation and apoptosis

**Fig. 5:**
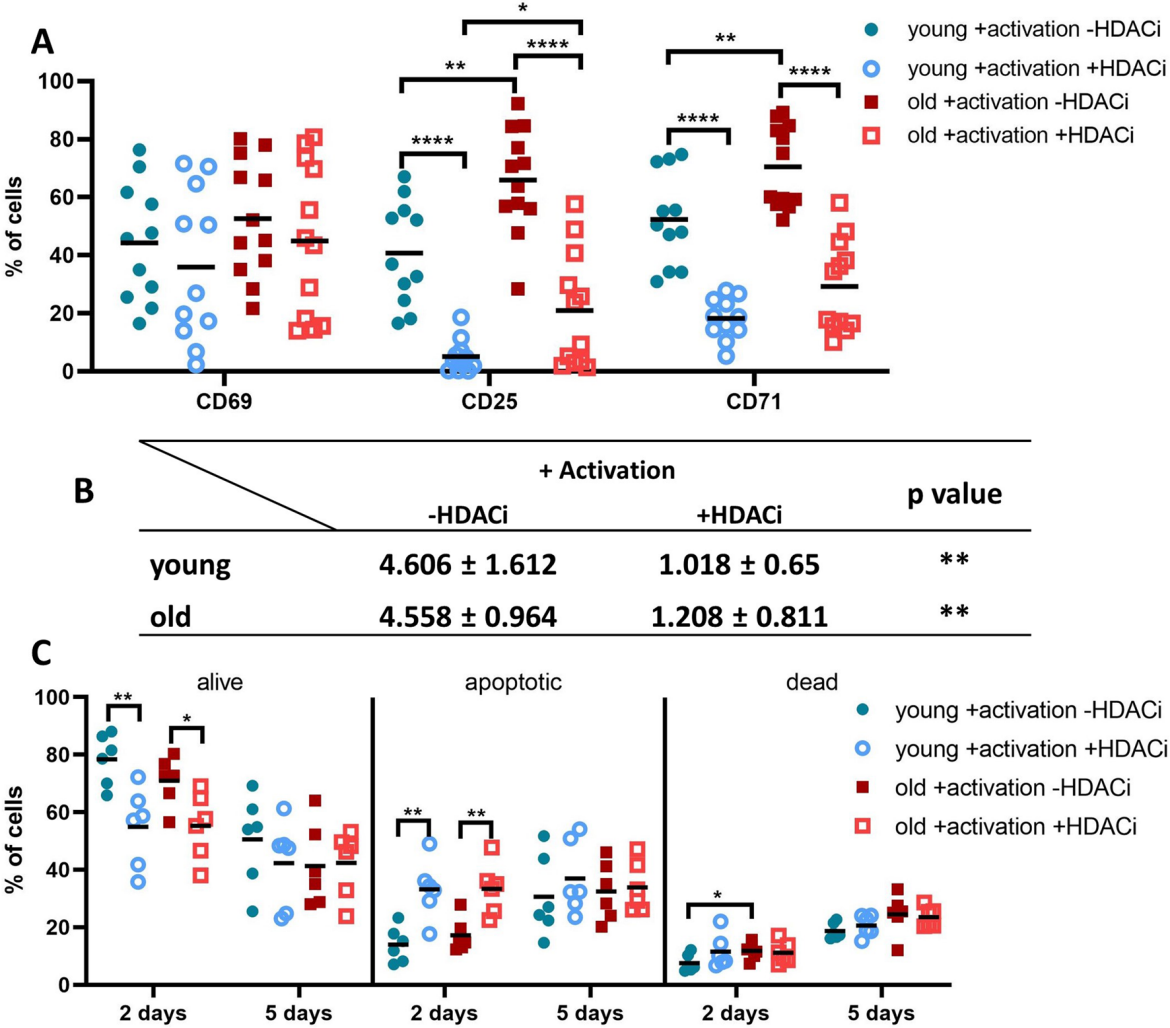
Activation markers, proliferation, and apoptosis of activated CD8^+^ T cells with and without HDACi treatment. **A** Differential expression of activation markers. The surface expression of common activation markers was measured by flow cytometry. The percentage of cells expressing the respective markers was obtained by gating the positive cells in the histogram of each marker; N young = 11, N old = 12. **B** Proliferation indexes after 5 days of culture. CD8^+^ T cells were stained with CFSE, treated as described and kept in culture for 5 days. The proliferation indexes were calculated with FCS Express 6 Plus Research Edition; N = 5. **C** Apoptosis assay after 2 and 5 days, respectively. The percentages of the cells that are alive (double negative), apoptotic (Annexin positive) and dead (double positive for Annexin and PI) are shown for both time points. N = 5. *p value < 0.05, **p value < 0.01, ****p value < 0.0001

To determine the functional effects of HDACi, the surface expression of CD69, CD25 and CD71 was analyzed in CD8^+^ T cells left untreated or treated for 2 days with HDACi (Fig. 5A). CD69 surface expression on CD8^+^ T cells was highly heterogeneous, but comparable within the two groups. In contrast, the expression of CD25 and CD71 was higher on the CD8^+^ T cells from old donors when compared to CD69. The expression of both markers was significantly decreased upon HDACi treatment with CD25 expression maintaining a significant difference between the two age groups. Since CD25 is the alpha chain of the IL-2 receptor [59] and CD71 a marker for proliferation [60], an increased proliferation and/or longer survival of CD8^+^ T cells from old donors was expected. However, there exists no difference in proliferation or apoptosis rates of CD8^+^ T cells between both age groups (Fig. 5B and C).

CD8^+^ T cells from both age groups divided on average 4.5–5 times during 5 days of culture and the presence of HDACi treatment drastically reduced the proliferation indexes of both groups (Fig. 5B). The apoptosis rates of untreated and HDACi treated CD8^+^ T cells from young and old donors were highly heterogeneous after long term treatment, while after 2 days of culture a difference was detected between untreated and treated cells from both age groups.

### Altered cytokine secretion patterns of CD8^+^ T cells during aging

**Fig. 6:**
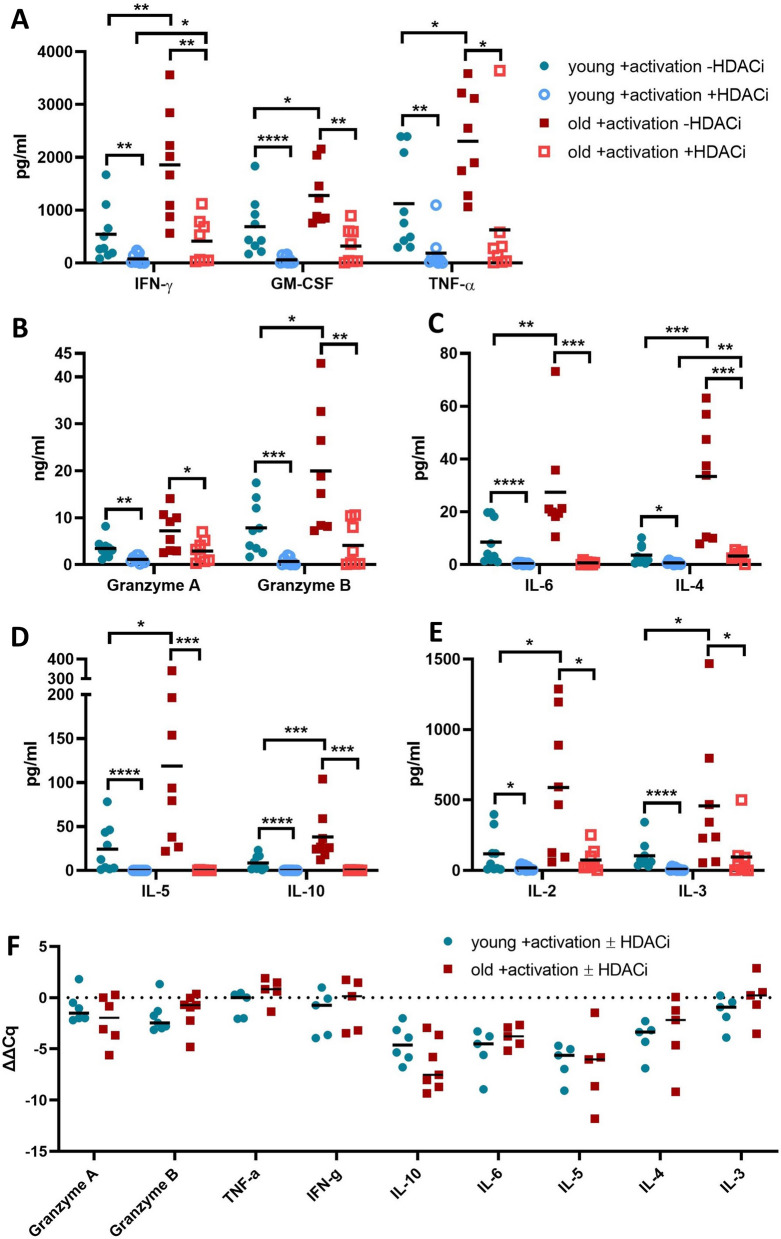
The effect of HDACi on the secretion machinery of activated CD8^+^ T cells. **A**–**E** Age-dependent cytokines´ secretion pattern upon HDACi treatment. CD8^+^ T cells were activated with aCD3/aCD28 mAb and then treated or not with HDACi. After 2 days in culture the secreted amounts of several cytokines and enzymes were determined in the supernatant. Granzyme A and B are the only ones with a different scale than the others. Results are presented as ng/ml and pg/ml. N young = 5, N old = 6. *p value < 0.05, **p value < 0.01. **F** Relative transcript expression for selected enzymes and cytokines. SDHA and TBP were used as housekeeper genes. N = 5–7

In order to determine the effect of HDACi on CD8^+^ T cell cytokine secretion pattern, a multiplex kit containing IL-2, IL-3, IL-4, IL-5, IL-6, IL-10, TNF-α, IFN-γ, GM-CSF, granzyme A and granzyme B was used to analyze their supernatants. Apart from granzyme A, untreated CD8^+^ T cells from old donors secreted significantly higher levels of the tested proteins (Fig. 6A–E). In line with the phenomenon of inflammaging [4, 61–66], CD8^+^ T cells from old individuals secreted more inflammatory cytokines, like TNF-α, IFN-γ and IL-6, than those from young donors, despite the secretion of IL-6 was never exceeding 100 pg/ml (Fig. 6C). Moreover, CD8^+^ T cells from old donors secreted much higher levels of IL-2 and IL-4 than that of young donors.

Although IL-2 is a known survival factor of T cells, the higher IL-2 secretion levels did not improve the proliferation or survival of CD8^+^ T cells from old donors. Whereas this age-dependent increase of IL-2 has also been observed in other studies [65, 66], reports on IL-4 are more contradictory [66, 67]. It is noteworthy that CD8^+^ T cells of old donors had in general a more heterogeneous secretion of cytokines than that of young donors.

The HDACi treatment strongly reduced the secretion of all soluble proteins tested for both groups, but did not completely abolish differences between ages. Whereas the CD8^+^ T cells from young donors were highly affected by the treatment with an almost total inhibition of the secretory machinery, the CD8^+^ T cells from old donors maintained a low level of secretion for most cytokines with a total loss of IL-10, IL-6 and IL-5 secretion. Using qPCR, the data were confirmed by a similar relative gene expression pattern of these cytokines (Fig. 6F).

## Discussion

Epigenetic processes including DNA methylation and histone modifications have been implicated in the progression of age-related disorders and cancers. Therapeutic targeting of these processes led to novel therapeutic strategies for the prevention and/or treatment of diseases. Indeed, HDACi have been successfully implemented in the treatment of diverse tumor entities [68]. Despite HDACi have been shown to rewire cancer-associated transcriptional programs and affect adaptive immunity [69], little information exists on the age-dependent role of HDACi on immune cells. Therefore, this study analyzed the interplay between acetylation and aging. First, CD8^+^ T cells were magnetically sorted from PBMC of young and old healthy blood donors and activated in vitro in the presence or absence of a HDACi cocktail [31, 70–73]. As shown in Fig. 1A–C, the relative protein and mRNA expression of HDAC in CD8^+^ T cells from the two age groups did not significantly differ. While SIRT1 has been shown to be decreased in aged tissues [74–76] and SIRT3 and 6 have been linked to longevity [77–80], no differences in the expression pattern of these genes were detected between the age groups within the current cohort. HDAC3 [16, 81] and HDAC4 [82, 83], which are required for post-thymic T cell maturation, have displayed statistical, but no biological significant differences in their gene expression between the age groups (Fig. 1A). HDAC11, a known negative regulator of immune cell functions [18], neither exhibited an age-dependent difference at the mRNA level, nor at a protein level (Fig. 1A and B). Furthermore, the acetylation profiles observed with WB were also similar for both age groups, despite one outlier within the old group (Fig. 1E). In order to determine whether there exist differences in the protein expression of activated CD8^+^ T cells from young and old donors, 2DE-based proteomics approach was implemented. Despite the fact that this method has specific technical limitations regarding the number of spots identified using a total, unfractionated lysate, more than 80% of the identified proteins had a decreased expression indicating a downregulation of the most abundant proteins in CD8^+^ T cells.

HDACi treatment resulted in the modification of several proteins in only one age group (2 for the old and 12 for the young donors—Additional file 1: Table S2). Only in old donors, TRUB1 and EF2 had a significantly increased or decreased expression, respectively. These two proteins are regulators of the mRNA processing [84, 85], with EF2 known as a target for cancer therapy [86].

Moreover, the proteins identified were not a random group, but were interconnected and participated in shared functions as demonstrated by the high protein–protein interaction enrichment found upon STRING analysis (< 10^–13^). As expected, most GO terms were associated to the immune system (Fig. 3) including terms specifically related to the inflammatory activity of CD8^+^ T cells.

A higher secretion of pro-inflammatory factors from CD8^+^ T cells of old donors is well-documented [4, 63–66]. In the current study, the secretion of CD8^+^ T cells from old donors in the absence of HDACi treatment is higher for all soluble proteins tested, including anti-inflammatory cytokines, such as IL-10 and IL-4. In some studies IL-10 was found to be increased with aging [87]. Although IL-10 is primarily secreted by CD4^+^ T cells, a subpopulation of CD8^+^ T cells with regulatory functions has been also shown to secrete high amounts of IL-10 [88–90]. In the current study, CD8^+^ T cells of old donors produced significantly more IL-10 than that of young donors suggesting that within the CD8^+^ T cell population of the old donors an increased frequency of cells with regulatory properties exists.

The HDACi treatment of CD8^+^ T cells induces a reduction of cytokine secretion in both age group, but to a different extent. While HDACi treatment induces a shut down in the secretion of the cytokines analyzed in the young group, the secretion of pro-inflammatory and cytotoxic factors is maintained in the old group. Since the transcript levels of these soluble proteins are mostly down-regulated (Fig. 6F) HDACi treatment blocked the production of these proteins and not only their exocytosis, which is probably a result of the reduced availability of the euchromatin due to an increased cellular acetylation [91]. In most cases, the decreased secretion was higher in CD8^+^ T cells from young donors. Thus, the anti-inflammatory effect of HDACi on CD8^+^ T cells is stronger in young individuals.

Additional to the cytokine secretion, several GO terms also referred to the activation and proliferation of the CD8^+^ T cells (Fig. 3 and Additional file 1: Table S3). So far, the evidence that the surface expression of activation markers is different between CD8^+^ T cells from individuals of different ages is limited [92, 93]. Therefore, the expression of CD69, CD25 and CD71 was determined on CD8^+^ T cells from old and young donors with and without HDACi treatment. A heterogeneous CD69 expression was found in CD8^+^ T cells in both age groups regardless of HDACi treatment, which is not in accordance with the literature [92, 93]. In contrast, both CD25 and CD71 have an age-dependent expression, which was severely decreased after HDACi treatment (Fig. 5A). The reduced activation caused by the HDACi treatment was associated with a decreased expression of p-AKT (Fig. 4A), which is indirectly involved in the TCR-mediated T cell activation by controlling the phosphorylation of Foxo1 [35]. Non-phosphorylated AKT was not altered by HDACi treatment in both age groups, while p-AKT had a lower expression in CD8^+^ T cells from young donors. Furthermore, HSP90 had a decreased expression in the 2D protein expression profiles (Additional file 1: Table S2) and the WB data (Fig. 2C and D) after the HDACi treatment for both age groups. HSP90 plays a critical role in eliciting both CD4^+^ and CD8^+^ T cell responses [94] by acting as a “docking site” for many kinases that are part of the TCR signaling pathway [95]. Furthermore, treatment of T cells with geldanamycin, a specific HSP90 inhibitor, has been shown to reduce the surface expression of glycoproteins, such as CD3, CD4 and CD8 [96].

The 14-3-3 ζ/δ protein, however, had an increased protein expression after HDACi treatment (Additional file 1: Table S2, Fig. 2C and D). The 14-3-3 protein family is involved in the TCR signaling pathway; they bind to phosphorylated FoxO transcription factors and block their activity, permitting the activation of T cells [35, 97]. FoxO transcription factors are more active in quiescent naïve T cells [36], which could explain the higher expression of the 14-3-3 ζ/δ protein in young individuals with a 1.62 difference in the RFs.

The higher expression levels of CD25 and CD71 on CD8^+^ T cells of old donors coupled with an increased secretion of IL-2 (Fig. 6) suggest that CD8^+^ T cells from older individuals might have an increased survival. However, no differences were found in the proliferation and apoptosis rates of CD8^+^ T cells from young and old donors. Furthermore, the proliferation of CD8^+^ T cells was similar between both age groups and equally inhibited by HDACi treatment (Fig. 5B). An effect of HDACi treatment on the apoptosis rate of CD8^+^ T cells was significant in the first 2 days in culture but disappears after 5 days (Fig. 5C). This is in contrast to other studies demonstrating that HDACi negatively interferes with the proliferation and induces apoptosis in cancer cell lines [31, 70–73]. In the current study, the effect on the apoptosis rate of CD8^+^ T cells was limited, while the T cell activation and proliferation was directly inhibited by this treatment.

In the network analysis, there were no GO terms found to be associated with the differentiation of CD8^+^ T cells, although the inhibitors used had effects on the cell differentiation in other studies [70–72, 98]. Additionally, transcripts implicated in CD8^+^ T cell differentiation [56, 57] were analyzed by qPCR in both groups (Fig. 4C) demonstrating a similar distribution between both age groups with the exception of CNR2, which was shown to be involved in the secretion of inflammatory cytokines [58]. Therefore, an effect of HDACi on the differentiation of CD8^+^ T cells cannot be postulated.

One highlight of the identified proteins were changes in the cytoskeleton (Additional file 1: Table S2), which is important for the T cell activation [99–102]. Numerous GO terms related to the cytoskeleton were found with most of the proteins identified localized in the cytoplasm, while some were distributed between “secretory granule lumen” and “intracellular non-membrane-bounded organelle” (Fig. 3F). The HDACi treatment reduced the expression of tubulin mRNA and protein (Fig. 4A) with a higher expression of acetyl-tubulin in CD8^+^ T cells from old donors (Fig. 4B).

## Conclusions

In our study, we have analyzed the effect of acetylation on the proteome of CD8^+^ T cells from young and old healthy blood donors by comparing the protein expression patterns of untreated and HDACi-treated CD8^+^ T cells. Using this approach, the intracellular acetylation levels were increased upon HDACi treatment. Furthermore, acetylation affects the proteome of CD8^+^ T cells in an organized manner, targeting primarily the cytoskeleton and cellular chaperones, which coincided with a significant reduction in secretion and proliferation after triggering the TCR of CD8^+^ T cells.

Upon comparison of the effect of HDACi on the activation of CD8^+^ T cells from young and old donors, CD8^+^ T cells from old donors had a stronger response to TCR-mediated activation as demonstrated by a higher surface expression of CD25 and CD71, a stable p-AKT expression, an increased secretion of IL-2 and an increase in acetyl-tubulin. Nevertheless, the HDACi treatment strongly affected T cell activation of both age groups.

It is noteworthy that HDACi compounds are currently used as anti-inflammatory and anti-cancerous agents in clinical settings. Our findings demonstrate a residual inflammatory activity for the CD8^+^ T cells from old donors after the HDACi treatment suggesting that the treatment efficacy is age-dependent, which must be considered for their clinical implementation. Since the data presented are exploratory and need to be confirmed in an independent cohort, the interplay of aging and HDACi treatment must be considered in the future and the role of acetylation in the aging immune system has to be investigated further.

## Supplementary Information

**Additional file 1: Table S1.** Primers for qPCR analysis. **Table S2.** Differentially expressed proteins of activated CD8^+^ T cells from young and old donors in the presence and absence of HDACi. **Table S3.** Classification of GO terms returned by STRING for the differentially expressed proteins.

## Data Availability

The datasets used and/or analyzed during the current study are available from the corresponding author on reasonable request.

## Abbreviations

CNR2: Cannabinoid receptor 2
CFSE: Carboxyfluorescein succinimidyl ester
CCR7: C–C chemokine receptor 7
HDAC: Deacetylase
DIGE: Difference gel electrophoresis
EF2: Elongation factor 2
FDR: False discovery rate
FoxO: Forkhead box O
GOI: Gene of interest
GO: Gene ontology
GAPDH: Glyceraldehyde-3-phosphate dehydrogenase
GM-CSF: Granulocyte–macrophage colony-stimulating factor
HSP90: Heat shock protein 90
HSP90A: Heat shock protein 90 alpha
HDACi: Histone deacetyalse inhibitor
HK: Housekeeper
KEGG: Kyoto encyclopedia of genes and genomes
SELL: L-selectin
MALDI-TOF MS: Matrix-assisted laser-desorption/ionization time-of-flight mass spectrometer
mTOR: Mechanistic target of rapamycin
mAb: Monoclonal antibody
mTORC2: MTOR complex 2
NAM: Nicotinamide
PBMC: Peripheral blood mononuclear cells
PTM: Posttranslational modifications
PI: Propidium iodide
AKT: Protein kinase B
Cq: Quantification cycle
qPCR: Quantitative PCR
RF: Regulation factor
STRING: Search tool for the retrieval of interacting genes
SIRT: Sirtuin
SDS-PAGE: Sodium dodecyl sulphate–polyacrylamide gel electrophoresis
SDHA: Succinyldehydrogenase subunit A
TCR: T cell receptor
TBP: TATA-binding box protein
TSA: Trichostatin a
TRUB1: TRNA pseudouridine synthase 1
2DE: Two-dimensional gel electrophoresis
tyrosine- PTN6: Protein phosphatase non-receptor type 6
WB: Western blot
