# Supplementary material for: Characterization of the effect of histone deacetylation inhibitors on CD8+ T cells in the context of aging
Source: J Transl Med. 2022 Nov 22;20:539. doi: 10.1186/s12967-022-03733-9 (PMC9682763; doi:10.1186/s12967-022-03733-9)
Supplement: Supplementary file 1 — Additional file 1: Table S1. Primers for qPCR analysis. Table S2. Differentially expressed proteins of activated CD8+ T cells from young and old donors in the presence and absence of HDACi. Table S3. Classification of GO terms returned by STRING for the differentially expressed proteins. [file 12967_2022_3733_MOESM1_ESM.docx]

# **Additional file 1:**

Table S1. Primers for qPCR analysis. All primers were ordered from Integrated DNA Technologies (Coralville, Iowa, U.S.A.) and dissolved in Ampuwa water to a final concentration of 10 mM *(Fw = forward, 5’–3’; Rw = reverse, 3’–5’).*

| Gene | Transcript | Sense | Sequence 5'→3' | Product length | Annealing temperature |
| --- | --- | --- | --- | --- | --- |
| *SIRT1* | NM_012238.5 | Fw | GGCGGCTTGATGGTAATCAGT | 148 | 60 |
|  |  | Rw | CTTGGACTCTGGCATGTCCC |  |  |
| *SIRT3* | NM_001370314.1 | Fw | GCTTCCTCTAGTGACACTGTTAG | 135 | 58 |
|  |  | Rw | TGCAGAAGTAGCAGTTCAGTG |  |  |
| *SIRT6* | NM_001321058.2 | Fw | CATCCTAGACTGGGAGGA | 172 | 56 |
|  |  | Rw | CAGGTTGACGATGACCAG |  |  |
| *HDAC1*(103) | NM_004964.3 | Fw | GGAAATCTATCGCCCTCACA | 168 | 58 |
|  |  | Rw | AACAGGCCATCGAATACTGG |  |  |
| *HDAC2*(103) | NM_001527.4 | Fw | TAAATCCAAGGACAACAGTGG | 89 | 58 |
|  |  | Rw | GGTGAGACTGTCAAATTCAGG |  |  |
| *HDAC3*(103) | NM_003883.4 | Fw | TAGACAAGGACTGAGATTGCC | 120 | 58 |
|  |  | Rw | GTGTTAGGGAGCCAGAGCC |  |  |
| *HDAC4*(103) | XM_024453257.1 | Fw | GGTTTATTCTGATTGAGAACTGG | 146 | 58 |
|  |  | Rw | ATTGTAAACCACAGTGCTCGC |  |  |
| *HDAC11*(104) | NM_001330636.2 | Fw | CAATGGGCATGAGCGAGAC | 68 | 60 |
|  |  | Rw | TGTGGCGGTTGTAGACATCC |  |  |
| *p300* | NM_001362843.2 | Fw | GGAAGTGCTGGCAACTTACTG | 125 | 58 |
|  |  | Rw | CCATAAGGATTGGGGTTGTTC |  |  |
| *Granzyme B* (105) | NM_001346011.2 | Fw | TGGGGGACCCAGAGATTAAAA | 100 | 58 |
|  |  | Rw | TTTCGTCCATAGGAGACAATGC |  |  |
| *Granzyme A* (106) | NM_006144.4 | Fw | TTTCTGGCATCCTCTCTCTCA | 305 | 58 |
|  |  | Rw | GGGTCATAGCATGGATAGGG |  |  |
| *TNF-a* | NM_000594.4 | Fw | GCTGCACTTTGGAGTGATCG | 215 | 56 |
|  |  | Rw | TATCTCTCAGCTCCACGCCA |  |  |
| *IFN g* | NM_000619.3 | Fw | GCTCTGCATCGTTTTGGGTT | 488 | 56 |
|  |  | Rw | ATTGCAGGCAGGACAACCAT |  |  |
| *IL-10* (107) | NM_000572.3 | Fw | CTGTGAAAACAAGAGCAAGGC | 500 | 58 |
|  |  | Rw | GAAGCTTCTGTTGGCTCCC |  |  |
| *IL-6* | NM_000600.5 | Fw | TTCGGTCCAGTTGCCTTCTC | 316 | 56 |
|  |  | Rw | TCACCAGGCAAGTCTCCTCA |  |  |
| *IL-5* | NM_000879.3 | Fw | AGCCAATGAGACTCTGAGGAT | 359 | 56 |
|  |  | Rw | AGGCCTGACTCTTTCTTGGC |  |  |
| *IL-4* | NM_000589.4 | Fw | TCTTTGCTGCCTCCAAGAACA | 235 | 56 |
|  |  | Rw | TCCAACGTACTCTGGTTGGC |  |  |
| *IL-3* | NM_000588.4 | Fw | CCTTTGCCTTTGCTGGACTTC | 335 | 56 |
|  |  | Rw | AGAGAACGAGCTGGACGTTG |  |  |
| *Sell* | NM_000655.5 | Fw | TTCAAGTTGTGGGGGTGGAC | 433 | 58 |
|  |  | Rw | GCTGGCAAGAAGCTGTGTAAC |  |  |
| *CNR2* | NM_001841.3 | Fw | TCAACAGGTGCTCTGAGTGG | 597 | 58 |
|  |  | Rw | CGGGTGAGCAGAGCTTTGTA |  |  |
| *SLFN5* | NM_001330183.2 | Fw | CCTTACGAGCTTGAGGGCTT | 406 | 58 |
|  |  | Rw | TGCTGAAACTGGAAAGTAGCG |  |  |
| *S1PR5* | NM_001166215.2 | Fw | CCTGCTAAGCAACCCACTGA | 260 | 58 |
|  |  | Rw | TCTCTAGCACGATGAAGGCG |  |  |
| *IL7R* | NM_002185.5 | Fw | CACGATGTAGCTTACCGCCA | 230 | 58 |
|  |  | Rw | ATAGGATCCATCTCCCCTGAGC |  |  |
| *BCL2* | NM_000633.2 | Fw | TCCAGGATAACGGAGGCTGG | 554 | 58 |
|  |  | Rw | GATGGTGATCCGGCCAACAA |  |  |
| *TBX21* | NM_013351.2 | Fw | CCACCTGTTGTGGTCCAAGT | 353 | 58 |
|  |  | Rw | GGAGCACAATCATCTGGGTCA |  |  |
| *TBP* | NM_003194.5 | Fw | CTGGCCCATAGTGATCTTTGC | 73 | 58 |
|  |  | Rw | TCAATTCCTTGGGTTATCTTCACA |  |  |
| *SDHA* | NM_004168.4 | Fw | TGGGAACAAGAGGGCATCTG | 86 | 58 |
|  |  | Rw | CCACCACTGCATCAAATTCATG |  |  |
| *Tubulin* | NM_006009.4 | Fw | CGCGCTGTAAGAAGCAACAA | 207 | 58 |
|  |  | Rw | TCTCCTCCCCCAATGGTCTT |  |  |
| *AKT* | NM_005163.2 | Fw | TGGTCCTGTCTTCCTCATGTT | 600 | 58 |
|  |  | Rw | TTGTCCACTCCTCCCGCT |  |  |
| *HIF1* | NM_001530.4 | Fw | CGGGGACCGATTCACCAT | 600 | 58 |
|  |  | Rw | GTGCAGTGCAATACCTTCCA |  |  |
| *NF-kB* | NM_001077494.3 | Fw | CGGGACAAGAGAAAAGAGGGA | 372 | 58 |
|  |  | Rw | ATCGGAAGCCTCTCTGCTTA |  |  |
| *HS90A* | NM_001017963.3 | Fw | CGTTTCTGAGAAGCAGGGCA | 285 | 60 |
|  |  | Rw | CGAACGTCTCAACCTCCTCC |  |  |
| *HS90B* | NM_003299.3 | Fw | TGTATGGAGCAGCAAGACTGA | 410 | 60 |
|  |  | Rw | ATACACGGCGCACATAGAGC |  |  |
| *IF4A1* | NM_001204510.2 | Fw | TCCTAGCACCCACTCGAGAA | 165 | 60 |
|  |  | Rw | CAGGGGTACCCACGATGATG |  |  |
| *XRCC5* | NM_021141.4 | Fw | AGCATAGACTGCATCCGAGC | 315 | 60 |
|  |  | Rw | TCCCCATACATCCACGACCT |  |  |
| *EF2* | NM_013302.5 | Fw | TGTGTTCCCTGATCACTCGT | 357 | 58 |
|  |  | Rw | CACTGTGTTTTGGTGCCCTG |  |  |
| *PLSL* | NM_002298.5 | Fw | ACCAGTACTACCAAGGACAGC | 495 | 60 |
|  |  | Rw | TTGGGATGACATGCCGACAA |  |  |
| *SFPQ* | NM_005066.3 | Fw | CTACATGGATCCACGGGAAAG | 600 | 58 |
|  |  | Rw | GGGCCTTCGTACTCTTCTCTC |  |  |

Table S2. Differentially expressed proteins of activated CD8^+^ T cells from young and old donors in the presence and absence of HDACi. A complete list of the identified proteins is presented. For each protein are given the molecular mass in kDa, the MOWSE score, the number of peptides that were matched to the protein sequence, the protein sequence coverage as a percentage and the calculated isoelectric point (pI). The regulation factors (RF) were calculated based on the 5 biological replicates for each age group as a ratio between the untreated and HDACi treated preparations. All proteins have RFs under 0.66 (green), which represent a decrease in expression after HDACi treatment, and RF over 1.5 (yellow), which represent an increase. The values of the RFs are presented only when they are within the predetermined cutoff. P values have been calculated with a T-test between the control and the HA sample for each group. * = p value < 0.05; ** = p value < 0.01; *** = p value < 0.001; **** = p value < 0.0001.

| Spot no. | UniProt Accession | UniProt entry name | Description | Mass (kDa) | Score | Matched peptides | Sequence coverage (%) | Calculated pI | RF young | RF old |
| --- | --- | --- | --- | --- | --- | --- | --- | --- | --- | --- |
| 1 | P63104 | 1433Z | 14-3-3 protein zeta/delta | 27.90 | 96 | 17 | 48 | 4.7 | 4.82**** | 3.17** |
| 2 | P14618 | KPYM | Pyruvate kinase PKM | 58.47 | 120 | 16 | 40 | 8.0 | 0.64** | 0.62 |
| 3 | P60842 | IF4A1 | Eukaryotic initiation factor 4A-I | 46.35 | 109 | 20 | 44 | 5.3 | 0.56** | 0.61* |
| 4 | P11142 | HSP7C | Heat shock cognate 71 kDa protein | 71.08 | 211 | 32 | 50 | 5.4 | 0.53*** | 0.52**** |
| 5 | P23246 | SFPQ | Splicing factor, proline- and glutamine-rich | 76.22 | 61 | 15 | 23 | 9.5 | 1.57** | 1.91 |
| 6 | P13010 | XRCC5 | X-ray repair cross-complementing protein 5 | 83.22 | 180 | 30 | 33 | 5.6 | 2.09** | 1.67 |
| 7 | P11142 | HSP7C | Heat shock cognate 71 kDa protein | 71.08 | 211 | 32 | 50 | 5.4 | 0.52**** | 0.53*** |
| 8 | P07900 | HS90A | Heat shock protein HSP 90-alpha | 85.01 | 56 | 15 | 21 | 4.9 | 0.47* | 0.42** |
| 9 | Q8NFD4 | YI018 | Uncharacterized protein FLJ76381 | 17.10 | 62 | 5 | 30 | 11.1 | 0.51*** | 0.46** |
| 10 | P29350 | PTN6 | Tyrosine-protein phosphatase non-receptor type 6 | 67.92 | 78 | 15 | 27 | 7.7 | 0.62** | 0.50** |
|  | P14866 | HNRPL | Heterogeneous nuclear ribonucleoprotein L | 64.72 | 66 | 16 | 27 | 8.5 |  |  |
| 11 | P08238 | HS90B | Heat shock protein HSP 90-beta | 83.55 | 205 | 34 | 38 | 5.0 | 0.42*** | 0.53*** |
|  | P07900 | HS90A | Heat shock protein HSP 90-alpha | 85.01 | 122 | 27 | 34 | 4.9 |  |  |
| 12 | P60842 | IF4A1 | Eukaryotic initiation factor 4A-I | 46.35 | 117 | 23 | 40 | 5.3 | 0.56** | 0.01 |
|  | Q14240 | IF4A2 | Eukaryotic initiation factor 4A-II | 46.60 | 86 | 19 | 38 | 5.3 |  |  |
| 13 | P60709 | ACTB | Actin, cytoplasmic 1 | 42.05 | 183 | 21 | 47 | 5.3 | 0.46**** | 0.42* |
|  | P63261 | ACTG | Actin, cytoplasmic 2 | 42.11 | 183 | 21 | 47 | 5.3 |  |  |
|  | P63267 | ACTH | Actin, gamma-enteric smooth muscle | 42.25 | 87 | 13 | 28 | 5.3 |  |  |
|  | P62736 | ACTA | Actin, aortic smooth muscle | 42.38 | 87 | 13 | 28 | 5.2 |  |  |
|  | P68032 | ACTC | Actin, alpha cardiac muscle 1 | 42.33 | 87 | 14 | 31 | 5.2 |  |  |
|  | P68133 | ACTS | Actin, alpha skeletal muscle | 42.37 | 86 | 14 | 31 | 5.2 |  |  |
| 14 | P13639 | EF2 | Elongation factor 2 | 96.25 | 95 | 21 | 24 | 6.4 |  | 0.57** |
| 15 | Q8WWH5 | TRUB1 | Probable tRNA pseudouridine synthase 1 | 37.52 | 56 | 8 | 28 | 8.4 |  | 2.02** |
| 16 | P13796 | PLSL | Plastin-2 | 70.81 | 233 | 33 | 48 | 5.3 | 0.52 |  |
| 17 | Q99714 | HCD2 | 3-hydroxyacyl-CoA dehydrogenase type-2 | 27.13 | 71 | 9 | 48 | 7.7 | 1.72*** |  |
| 18 | O43707 | ACTN4 | Alpha-actinin-4 | 105.25 | 97 | 21 | 22 | 5.3 | 1.54* |  |
| 19 | Q9C0H5 | RHG39 | Rho GTPase-activating protein 39 | 122.24 | 65 | 14 | 13 | 7.3 | 0.65** |  |
| 20 | P52597 | HNRPF | Heterogeneous nuclear ribonucleoprotein F | 45.99 | 129 | 17 | 47 | 5.4 | 0.58**** |  |
| 21 | P08670 | VIME | Vimentin | 53.68 | 123 | 24 | 35 | 5.1 | 0.62**** |  |
| 22 | P07437 | TBB5 | Tubulin beta chain | 50.10 | 185 | 27 | 48 | 4.8 | 0.64** |  |
|  | Q13885 | TBB2A | Tubulin beta-2A chain | 50.27 | 133 | 22 | 37 | 4.8 |  |  |
|  | Q9BVA1 | TBB2B | Tubulin beta-2B chain | 50.38 | 133 | 22 | 37 | 4.8 |  |  |
|  | P68371 | TBB4B | Tubulin beta-4B chain | 50.26 | 144 | 24 | 42 | 4.8 |  |  |
|  | P04350 | TBB4A | Tubulin beta-4A chain | 50.01 | 119 | 22 | 38 | 4.8 |  |  |
|  | Q13509 | TBB3 | Tubulin beta-3 chain | 50.86 | 107 | 20 | 30 | 4.8 |  |  |

Table S3. Classification of GO terms returned by STRING for the differentially expressed proteins. Sixty-nine of the total GO terms retrieved by STRING are presented below. The observed gene count represents the proteins observed in the input which are annotated with a certain term. The background gene count represents the total number of genes that are annotated with a certain term. The strength describes the size of the enrichment effect. The false discovery rate (FDR) is a p value adjusted for multiple testing.

| GO term | Term description | Observed gene count | Background gene count | Strength | FDR |
| --- | --- | --- | --- | --- | --- |
| Biological process | | | | | |
| GO:0007010 | cytoskeleton organization | 13 | 953 | 0.91 | 1.49E-06 |
| GO:0002252 | immune effector process | 12 | 927 | 0.89 | 8.84E-06 |
| GO:0002366 | leukocyte activation involved in immune response | 10 | 616 | 0.98 | 1.71E-05 |
| GO:0006996 | organelle organization | 19 | 3131 | 0.56 | 1.71E-05 |
| GO:0043312 | neutrophil degranulation | 9 | 485 | 1.04 | 1.71E-05 |
| GO:0045055 | regulated exocytosis | 10 | 691 | 0.93 | 1.71E-05 |
| GO:0002253 | activation of immune response | 7 | 393 | 1.02 | 0.00016 |
| GO:0038096 | Fc-gamma receptor signaling pathway involved in phagocytosis | 4 | 73 | 1.51 | 0.00031 |
| GO:0019221 | cytokine-mediated signaling pathway | 8 | 655 | 0.86 | 0.00035 |
| GO:0042267 | natural killer cell mediated cytotoxicity | 3 | 26 | 1.84 | 0.00047 |
| GO:0030029 | actin filament-based process | 7 | 493 | 0.93 | 0.00048 |
| GO:0051973 | positive regulation of telomerase activity | 3 | 36 | 1.69 | 0.00098 |
| Molecular Function | | | | | |
| GO:0005200 | Structural constituent of cytoskeleton | 10 | 106 | 1.75 | 6.63E-13 |
| GO:0000166 | Nucleotide binding | 21 | 2097 | 0.77 | 3.83E-11 |
| GO:0003723 | RNA binding | 11 | 850 | 0.88 | 1.10E-06 |
| GO:0005525 | GTP binding | 8 | 366 | 1.11 | 1.69E-06 |
| GO:0005524 | ATP binding | 13 | 1462 | 0.72 | 2.82E-06 |
| GO:0003924 | GTPase activity | 7 | 283 | 1.17 | 3.72E-06 |
| GO:0031625 | Ubiquitin protein ligase binding | 7 | 311 | 1.13 | 6.21E-06 |
| GO:0016787 | Hydrolase activity | 15 | 2448 | 0.56 | 2.13E-05 |
| GO:0016887 | ATPase activity | 7 | 392 | 1.02 | 2.33E-05 |
| GO:0003725 | Double-stranded RNA binding | 4 | 70 | 1.53 | 4.31E-05 |
| GO:0030235 | Nitric-oxide synthase regulator activity | 2 | 6 | 2.3 | 0.0004 |
| Cellular Component | | | | | |
| GO:0005829 | cytosol | 26 | 4958 | 0.49 | 2.68E-08 |
| GO:0034774 | secretory granule lumen | 10 | 323 | 1.26 | 2.68E-08 |
| GO:0043232 | intracellular non-membrane-bounded organelle | 23 | 4005 | 0.53 | 4.58E-08 |
| GO:0005856 | cytoskeleton | 16 | 2068 | 0.66 | 9.04E-07 |
| GO:0032991 | protein-containing complex | 22 | 4792 | 0.43 | 5.91E-06 |
| GO:1990904 | ribonucleoprotein complex | 10 | 770 | 0.89 | 5.91E-06 |
| Reactome pathways | | | | | |
| HSA-168256 | Immune System | 22 | 1925 | 0.83 | 1.49E-12 |
| HSA-389977 | Post-chaperonin tubulin folding pathway | 5 | 20 | 2.17 | 1.00E-08 |
| HSA-69275 | G2/M Transition | 8 | 189 | 1.4 | 1.65E-08 |
| HSA-194315 | Signaling by Rho GTPases | 9 | 402 | 1.12 | 2.01E-07 |
| HSA-1280215 | Cytokine Signaling in Immune system | 10 | 654 | 0.96 | 6.95E-07 |
| HSA-5336415 | Uptake and function of diphtheria toxin | 3 | 6 | 2.47 | 2.74E-06 |
| HSA-168249 | Innate Immune System | 11 | 1012 | 0.81 | 3.49E-06 |
| HSA-449147 | Signaling by Interleukins | 8 | 439 | 1.03 | 3.78E-06 |
| HSA-162582 | Signal Transduction | 16 | 2605 | 0.56 | 7.15E-06 |
| HSA-2132295 | MHC class II antigen presentation | 5 | 119 | 1.4 | 1.05E-05 |
| HSA-2565942 | Regulation of PLK1 Activity at G2/M Transition | 4 | 85 | 1.45 | 6.26E-05 |
| HSA-9020591 | Interleukin-12 signaling | 3 | 47 | 1.58 | 0.00031 |
| HSA-8953854 | Metabolism of RNA | 7 | 652 | 0.8 | 0.00035 |
| HSA-1280218 | Adaptive Immune System | 7 | 733 | 0.75 | 0.0007 |
| KEGG pathways | | | | | |
| hsa05130 | Pathogenic Escherichia coli infection | 9 | 53 | 2 | 5.09E-14 |
| hsa04145 | Phagosome | 8 | 145 | 1.51 | 5.47E-09 |
| hsa04540 | Gap junction | 6 | 87 | 1.61 | 2.61E-07 |
| hsa04520 | Adherens junction | 4 | 71 | 1.52 | 0.00015 |
| hsa04141 | Protein processing in endoplasmic reticulum | 4 | 161 | 1.17 | 0.0022 |
| hsa04612 | Antigen processing and presentation | 3 | 66 | 1.43 | 0.0024 |
| hsa00620 | Pyruvate metabolism | 2 | 39 | 1.48 | 0.0142 |
| hsa04390 | Hippo signaling pathway | 3 | 152 | 1.07 | 0.0142 |
| hsa04921 | Oxytocin signaling pathway | 3 | 149 | 1.08 | 0.0142 |
| hsa05110 | Vibrio cholerae infection | 2 | 48 | 1.39 | 0.0156 |
| hsa05164 | Influenza A | 3 | 168 | 1.02 | 0.0156 |
| UniProt keywords | | | | | |
| KW-0007 | Acetylation | 29 | 3335 | 0.71 | 1.10E-16 |
| KW-0547 | Nucleotide-binding | 20 | 1758 | 0.83 | 7.43E-12 |
| KW-0488 | Methylation | 15 | 959 | 0.97 | 2.69E-10 |
| KW-0558 | Oxidation | 6 | 30 | 2.07 | 5.08E-10 |
| KW-0206 | Cytoskeleton | 15 | 1200 | 0.87 | 3.52E-09 |
| KW-0963 | Cytoplasm | 26 | 4979 | 0.49 | 3.52E-09 |
| KW-0225 | Disease mutation | 19 | 2968 | 0.58 | 3.20E-07 |
| KW-0067 | ATP-binding | 13 | 1367 | 0.75 | 1.59E-06 |
| KW-0342 | GTP-binding | 7 | 338 | 1.09 | 1.24E-05 |
| KW-0832 | Ubl conjugation | 15 | 2381 | 0.57 | 1.86E-05 |
| KW-0597 | Phosphoprotein | 26 | 8067 | 0.28 | 8.08E-05 |
| KW-0702 | S-nitrosylation | 3 | 55 | 1.51 | 0.00076 |
| KW-0143 | Chaperone | 4 | 196 | 1.08 | 0.0019 |
| KW-0648 | Protein biosynthesis | 3 | 128 | 1.14 | 0.0069 |
